# Universal Health Coverage and Facilitation of Equitable Access to Care in Africa

**DOI:** 10.3389/fpubh.2019.00102

**Published:** 2019-04-26

**Authors:** N'doh Ashken Sanogo, Arone Wondwossen Fantaye, Sanni Yaya

**Affiliations:** ^1^Interdisciplinary School of Health Sciences, Faculty of Health Sciences, University of Ottawa, Ottawa, ON, Canada; ^2^School of International Development and Global Studies, Faculty of Social Sciences, University of Ottawa, Ottawa, ON, Canada

**Keywords:** universal health coverage, equity, access to care, health care reform, global health, Africa

## Abstract

**Background:** Universal Health Coverage (UHC) is achieved in a health system when all residents of a country are able to obtain access to adequate healthcare and financial protection. Achieving this goal requires adequate healthcare and healthcare financing systems that ensure financial access to adequate care. In Africa, accessibility and coverage of essential health services are very low. Many African countries have therefore initiated reforms of their health systems to achieve universal health coverage and are advanced in this goal. The aim of this paper is to examine the effects of UHC on equitable access to care in Africa.

**Methods:** A systematic review guided by the Cochrane Handbook was conducted and reported in accordance with the Preferred Reporting Items for Systematic Reviews and Meta-Analyses criteria (PRISMA). Studies were eligible for inclusion if 1- they clearly mention studying the effect of UHC on equitable access to care, and 2- they mention facilitating factors and barriers to access to care for vulnerable populations. To be included, studies had to be in English or French. In accordance with PRISMA guidelines, our systematic review was registered with the International Prospective Register of Systematic Reviews (PROSPERO) on April 24, 2018 (registration number CRD42018092793).

**Results:** In all 271 citations reviewed, 12 studies were eligible for inclusion. Although universal health coverage seems to increase the use of health services, shortages in human resources and medical supplies, socio-cultural barriers, physical inaccessibility, lack of education and information, decision-making power, and gender-based autonomy, prenatal visits, previous experiences, and fear of cesarean delivery were still found to deter access to, and use of, health services.

**Discussion:** Barriers to greater effectiveness of the UHC correspond to various non-financial barriers. There are no specific recommendations for these kinds of barriers. Generally, it is important for each country to research and identify contextual uncertainties in each of the communities of the territory. Afterwards, it will be necessary to put in place adapted strategies to correct these uncertainties, and thus to work toward a more efficient system of UHC, resulting in positive impacts on health outcomes.

## Introduction

Set in 2015, the UN sustainable development goals (SDGs) present an opportunity for the international community to continue its commitment to improve health, which is a central component of development. Achieving universal health coverage (UHC), which refers to access to quality health-care services coverage and financial risk protection for all residents, is a key health target (SDG 3.8) in the SDGs (1). UHC is a significant objective for equitable and sustainable health outcomes worldwide and thereby a key path to promote progress toward other health-related SDG targets (1). Unfortunately, access to quality health services continues to be a problem for most people in the developing world (2). About 400 million people do not have access to basic quality health services globally, and among people living in low and middle-income countries, 6% experience extreme poverty as a result of payment for health services (2). In Africa, accessibility and coverage of essential health services are especially low. In fact, only 43% of pregnant women attended the four recommended prenatal visits compared to the global average of 55% as of 2014 (3, 4). Only 49% of births are attended by skilled health personnel compared to the global average of 70% (3, 4). Relative to financial risk protection, direct payments have been identified as a major cause of this situation across the continent, with several studies showing that direct payments of care provide limited access to care for the underprivileged and women (5–7).

In order to achieve UHC, adequate provision of healthcare as well as healthcare financing systems that ensure access to adequate care regardless of ability to pay were identified as significant needs (8). In 2005, the World Health Assembly of the WHO advocated to member states to aim for UHC and access to promotive, preventive, curative, rehabilitative, and palliative health interventions on the foundation of equity (3, 9). The path advocated by the WHO to achieve this UHC included prepayment of health care by significantly reducing direct payments and user fees (10, 11). Such an objective was believed to be achieved by a broader and more equitable tax system, a compulsory health insurance, or both (10). Today, the progress toward achieving SDG target 3.8 on UHC by 2030 is monitored through two major indicators: the coverage of essential health services, such as curative care and health promotion; the proportion of households with large expenditures on health from total income (12).

The WHO has identified health system strengthening, which refers to the improvement of a system's performance, as a major means to progress toward UHC (1). A functional health system is one that is organized around stakeholders associated with improving, maintaining, or restoring the health of their populations. Health system strengthening and UHC are interlinked with other goals and contribute to SDGs in numerous ways: reducing poverty (SDG1); equitable health outcomes and wellbeing and promoting global public health security (SDG3); improving the quality of education (SDG4); promoting gender equality (SDG 5); developing inclusive economic growth and decent jobs; and promoting inclusive societies (1). For progress of UHC and to ensure equitable access to UHC, the ability of services to reach all populations (population coverage), the availability of services that can be provided (service availability), and the extent to which individuals are protected from the financial consequences of accessing and receiving healthcare must be tracked, measured and tackled worldwide (13).

Since the early 2000s, many African countries began initiating reforms of their health systems to achieve universal health coverage and are still working toward this goal today. However, implementing UHC has been riddled with various challenges across the continent and there are uncertainties as to whether supposed UHC in African countries has been able to provide equitable access to healthcare for its populations, especially in deprived communities. As a result, the aim of this systematic review is to examine the effects of UHC in facilitating equitable access to care in Africa amongst underprivileged individuals and communities. For the purpose of a research inquiry with a specific scope, the review focuses on vulnerable populations, which are the most excluded individuals and groups from access to, and use of, health services. This will help inform whether and in which contexts UHC programs have been successful in increasing equitable access to care, as well as to provide guidance for future evidence-based healthcare reforms. To the best of our knowledge, no systematic reviews of the effect of UHC in facilitating equitable access to care in Africa currently exist.

## Methods

A systematic review guided in part by the Cochrane Handbook was conducted and reported in accordance with the Preferred Reporting Items for Systematic Reviews and Meta-Analyses criteria (PRISMA) (14, 15). In accordance with PRISMA guidelines, our systematic review was registered with the International Prospective Register of Systematic Reviews (PROSPERO) on April 24, 2018 (registration number CRD42018092793).

### Search Strategy

The research strategy protocol was developed in collaboration with a university Health Sciences Librarian to refine our queries and characterize them in terms of the elements [Population, Intervention, Comparison, and Outcomes (PICO)] used for the questions even if all these elements have not been used in the formal research strategy.

Search terms designed for Medline (see Appendix 1) and other databases included the following terms, namely: universal health coverage, health services accessibility, health equity, Africa. The following databases were searched without language restriction: MEDLINE, EMBASE, CINAHL, Global Health, and the Gray Literature. The PROSPERO registry was also searched for ongoing or recently completed pertinent systematic reviews. Reference lists of qualifying studies were also scanned. Citations published since the commitment of the WHO member countries in 2005 to reach Universal Health Coverage were searched (from 2005 to March 6, 2018).

### Selection Process

Studies were eligible for inclusion if 1- they clearly mention studying the effect of UHC on equitable access to care, and 2- they mention facilitating factors and barriers to access to care for vulnerable and underprivileged populations (the needy). To be included, studies had to be in English or French (Table 1).

**Table 1:** Systematic review inclusion and exclusion criteria.

	**Inclusion Criteria**	**Exclusion Criteria**
Study Design	Regardless of methodology, qualifying studies: effect of Universal Health Coverage on equitable access to primary healthcare for vulnerable populations (indigent) in Africa; focused on factors influencing access to healthcare for vulnerable populations.	Letters, editorials, and narrative reviews will be excluded.
Participants	All studies addressing vulnerable populations' access to health care in Africa	Studies addressing access to health care by non-vulnerable populations in Africa; access to health care by vulnerable or non-vulnerable populations in high-income or developed countries.
Language	Studies reported in English and French	Other languages than English and French
Publication date	Jan 2005 to March 6, 2018	Before Jan 2005

After identification of studies, duplicates were removed using standard software (Endnote 7). Two independent reviewers conducted two levels of screening after exporting references from ENDNOTE 7 to Covidence. Level one screening, using citation titles and abstracts, was to determine study relevance to the overall objective of the systematic review (Table 1). Level two screening of full text was to determine if citations met inclusion criteria. The two reviewers independently extracted data with disagreement resolved through discussion.

Study findings were extracted using a data extraction form that was initially pilot tested on three randomly selected included studies before its actual use. The two reviewers used the form to extract data independently.

## Method of Synthesis

The method of synthesis used for this review is a narrative synthesis as guided by Popay et al. (16). The method is ideal and renowned for synthesizing evidence from a range of diverse sources, including quantitative, qualitative and mixed-methods primary research. The tool used to synthesize findings is a textual narrative description, which enables a simple and structured manner for describing and summarizing primary research data (Popay).

### Comprehensiveness of Reporting

All included studies were appraised by both reviewers using the Critical Appraisal Skills Program (CASP) tool (17). The CASP tool has different tools for different study types (qualitative, quantitative, reviews, RCTs, etc.), so we used the appropriate tool depending on the study appraised. The appraisal tool included for each type of study between 10 and 12 questions and each question included options “Yes,” “No,” or “Cannot tell.” We considered that “Yes,” meant that the study included the subject of the evaluation of the question, and “No” and “Cannot tell” meant that the study did not include the subject of evaluation of the question. At the end of the evaluation of each of the 12 included studies, we gave a score. For example, if the study had 6 “Yes” and 4 “No—Cannot tell,” we gave it the score of 6/10 (60%). The two reviewers tested each study independently with the CASP guidelines regarding their design. Disagreements were resolved by consensus. Studies were considered high quality if they scored 80% or above of CASP criteria, medium quality if they scored 60–79.9% of CASP criteria, and low quality if they scored < 60% of CASP criteria.

### Data Analysis

The data were synthesized by classifying different study types. Due to heterogeneity across study outcomes, data were analyzed descriptively. Study comparisons were grouped to answer research questions, and findings were synthesized based on outcomes. Characteristics of included studies were analyzed descriptively, and results were presented in a narrative format recommended by PRISMA and Popay et al. (16).

## Results

**Figure 1:**
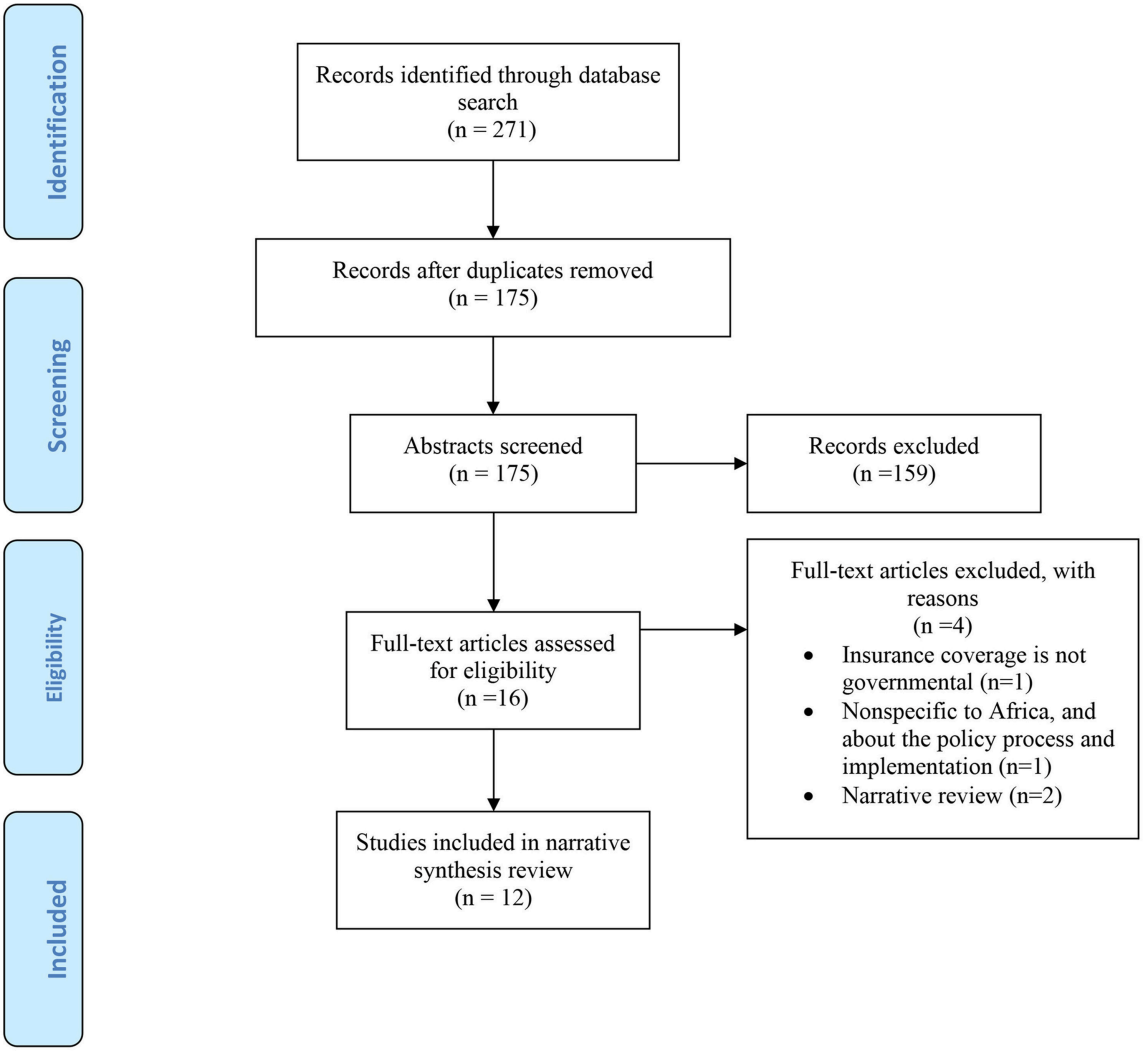
Flow diagram of study selection process.

Of the 271 citations reviewed, 12 studies published in 12 papers were eligible for inclusion (Figure 1). In terms of exclusions, duplicates were removed (*n* = 96), titles and abstracts not matching criteria (*n* = 159), and full texts that did not meet the inclusion criteria (*n* = 4) (see Appendix 2)

The 12 studies were published between 2005 and 2018, with most published in 2017. Studies were conducted in: Ghana (*n* = 6), Kenya (*n* = 2), Malawi (*n* = 1), Madagascar (*n* = 1), Burkina Faso (*n* = 1), and Rwanda (*n* = 1) (see Table 2).

**Table 2:** Characteristics of included studies.

**References**	**Location**	**Aim/Purpose**	**Design**	**Participants**	**Data source**	**Quality**	**Key findings**
Amporfu (8)	Ghana	This paper examines the vertical and horizontal equity of the premium collection scheme and the effect on the use of health services	Quantitative: Kakwani index method and graphical analysis	24.9 million adults	Self-administered structured questionnaires on National Health Insurance Scheme members randomly selected from the two main cities in the country (Accra and Kumasi	High	Revenue collection was vertically and horizontally inequitable
Aryeetey et al. (18)	Ghana	This paper analyses the costs and evaluate the equity, efficiency and feasibility of four strategies to identify poor households and their use of health services	Quantitative: households per setting with means testing as gold standard strategy	145–147 households	Household survey in the central region of Ghana in June 2009	High	Means testing was most efficient and equitable in rural and urban settings with low poverty incidence; the use of health services increases when fees are removed
Awoonor-Williams et al. (9)	Ghana	This paper aims to get a better understanding of how Ghana health insurance institutions interact with stakeholders and the effect in the use of primary health care	Mixed methods: Qualitative and survey methods	Various stakeholders in six selected districts	Semi-structured interviews	Medium	For optimal use of primary health services, some imperfections have to be fixed (delays in reimbursements of claims for services provided by health care providers, inadequate coordination among stakeholders in primary healthcare delivery)
Bonfrer et al. (19)	Ghana	This paper aims to evaluate the effects of Ghanaian Nation Health Insurance Scheme on maternal and infant healthcare use	Quantitative: statistical analysis	4,916 women aged 15–49	Household survey	High	All types of health care utilization included in this study is significantly higher among the insured. The scheme significantly increased the proportion of pregnancies with at least four ANC, and significant effect on attended deliveries
Campbell et al. (20)	Brazil, Ghana, Mexico, Thailand	This paper explores the policy lessons on human resources for health (HRH) from four countries that have achieved sustained improvement in UHC	Analytical framework: Case studies (qualitative)	Human resources for health	Administrative data	Medium	HRH are critical to the expansion of health service coverage and the package of benefits, so users can be confident
Chirwa et al. (21)	Malawi	This paper examined the features of services level agreements (SLA) and their effectiveness in expanding universal coverage.	Mixed methods	155 clients	Interviews and surveys	Medium	SLA improves the use of health services, particularly for the vulnerable and underserved populations. However, various factors (lack of clear guidelines, late payment of bills, lack of transparency, inadequate human and material resources) affect SLAs performance
Dalinjong,et al. (2)	Ghana	This paper examines the association between health insurance status and utilization of outpatient and inpatient health services in rural poor communities	Mixed methods: cross-sectional household survey and interviews	11,175 households 55,992 household members	Interviews and surveys	High	Being insured is associated with increased utilization of outpatient and inpatient health services in the study area
Garchitorena et al. (22)	Madagascar	This paper evaluates the impact of fee exemption in a rural area of Madagascar	Quantitative sectional household survey	1,522 households	Population and health system data	High	One-third of the needy have access to health care when fees are in place. However, when fees were removed, the use of health care increased by 65% for all patients, 52% for children under five and over 25% for maternity consultations
Kouanda et al. (23)	Burkina Faso	This paper aims to identify factors associated with home births in the Kaya health district where child delivery was free of charge	Mixed methods: interviews and statistical analysis	60,587 persons i.e., 8,825 households	Semi-structured interviews and administrative data	High	Key factors associated with home birth were age, distance from the household to the primary health centers, previous experience of giving birth at home, negative experiences with health centers, fear of cesarean delivery, and lack of transport.
Nyandekwe et al. (24)	Rwanda	This paper aims to assess Rwanda UHC	Quantitative: SWOT analysis	Rwanda national population	SWOT analysis from national retrospective review applied to six metrics as key indicators of UHC achievement related to WHO definition	High	96.15% of overall health insurance coverage, 1.07 visit per capita per year vs. 1 visit recommended by WHO. Rwanda UHC achievements are objectively convincing
O'Connell et al. (25)	Ghana, Bangladesh, Vietnam, Rwanda	This paper focuses on exploring the nature and extent of non-financial access barriers to care	Mixed methods: Literature review and household survey data	Published literature and household survey data	Surveys and literature	Medium	Barriers: ethnicity, religion, physical accessibility, decision-making, gender and autonomy, and knowledge, information
Were et al. (26)	Kenya	This paper evaluates the association of health insurance with access and utilization of obstetric delivery services.	Quantitative statistical analysis	4,082 pregnant women	Demographic and Health Survey	High	Mothers with insurance have 23% points more likely to deliver at an institution and 20% more likely to have access to skilled birth attendants compared to those not insured

### Quality Appraisal

The checklists cover the appropriateness of the research design, ethical considerations and standard conceptions for assessing risk of bias and overall quality. Quality assessment helped gather the relative strengths and weaknesses of the body of evidence.

The 12 included studies (6 mixed methods studies, 5 quantitative approach and 1 qualitative approach) demonstrated that their research designs and recruitment strategy were appropriate for addressing the aims of their research. Ethical issues were taken into consideration in only 5 out of the 12 included studies, with the rest not clearly demonstrating the maintenance of ethical standards or indicating ethical approval from committee. Although the 12 studies had a sufficiently rigorous data analysis process and a clear statement of findings, using multiple strategies to establish credibility, 4 of them did not discuss potential bias and limitation. No study was excluded due to assessment of conduct (validity and robustness), assessment of reporting (transparency) or assessment of content and utility of findings. The average overall quality is 8.3/10, which suggests the sum quality of the included studies for this review is high. Studies were not excluded or weighted based on the quality of the assessment; instead, the quality is used to inform data interpretation and ultimately determine the credibility of review findings and conclusions.

### Synthesis

As measured by health system reported service use, the use of health services by the poorest of the population increases equitable access to care in Africa, and this makes universal health coverage effective (19, 21, 22, 24, 26).

Rwanda is the most advanced country in Africa regarding universal coverage. The country has achieved 96.15% coverage in health insurance, with 1.07 patient health center visits per year as compared to 1 visit recommended by WHO (24). In addition, equity is demonstrated with 24.8% of the subsidized indigents vs. 24.1% living in extreme poverty, and they have access to quality services that meet WHO standards (24). Life expectancy increased from 49.74 years in 2001 to 68.21 in 2017.

In Madagascar, exemption from payment of health care in rural areas has led to an increase in the use of health services (22). In fact, less than one-third of people in need of care consulted in the past; but once the fees were removed, the use increased by 65% for all patients, 52% for children under five and more than 25% for maternity visits for an average direct cost of US $ 0.60 per patient (22). In Ghana, the government health insurance scheme increased the proportion of pregnancies with at least four antenatal visits by 7 points, with a significant effect on assisted deliveries by 10 points during the first year of operation (2, 19).

Although universal health coverage seems to have an effect on increasing the use of health services, some imperfections remain. One of these factors is the shortage and inequitable distribution of health professionals (20, 21, 23). In fact, achieving universal health coverage involves distributing resources, especially human capital for health, to match population needs. In Malawi, the shortage of health care providers and materials combined with various factors including late bill payments and lack of transparency have greatly affected their system's performance (21). Moreover, between 1990 and 2009, Ghana witnessed a rapid increase in its supply of professional health workers: 185% more midwives, 260% more nurses and 1300% more physicians (8, 20). Approximately 14,000 additional professional health workers were trained and employed, a number representing four times the increase in population growth (240 vs. 59%) over the same period (20). A strategic plan for the equitable distribution of health professionals in Ghana was set up to improve the decentralization of human capital for health across the country (20). In Rwanda, the situation is similar; equity in the distribution of health professionals throughout the national territory is respected (24). However, in Ghana, a study shows that key areas of misalignment between the operations of the national health insurance and that of primary health care was the delays in reimbursements of claims for services provided by health care provider; which serves as a demotivation for service providers (9).

The higher the health insurance coverage rate for the underprivileged, the more that people have access to it, displaying equity and social justice. In Madagascar, free reproductive health care has increased the use of services, including greater access to these services for the needy and thereby socially inclusive (22). In Rwanda, social justice exists to the extent that poor people and people living in extreme poverty have access to primary health services (as well as the more advantaged social classes) according to WHO standards (24). The Ghanaian health insurance has significantly increased access to prenatal care, and deliveries in health facilities for the most disadvantaged classes (19). However, although health insurance coverage increases the access of all social classes to primary health services by significantly reducing the financial contribution of users, the quality of its services (services capacity) still poses a problem in the majority of cases (20, 21, 23). There are governance issues of health systems. For instance, in Malawi, the lack of health professionals and their unequal distribution throughout the country, the stock-outs of medicines and health care equipment, as well as the late payment of rebate payments to health professionals, lead to a poor takeover of patients and demotivation of health professionals (21). In this case, even if the populations have access, the poor quality of the services will lead to a lack of confidence of the patients and a decrease of the use of the primary health services. Equity will not be respected because the wealthiest will go to private clinics or out of their country and the poor will be left to their own fate. As mentioned earlier, Ghana and Rwanda are success stories in this area as they have a strategic plan for the governance of their health systems according to WHO standards (20, 24).

Finally, aside from financial risk coverage and quality of care, other factors are important to consider in order to ensure equity and performance of health systems (22). This includes ethnicity, religion, physical accessibility, decision-making, gender and autonomy, information and education. These non-financial factors pose considerable barriers to access because they are mostly sociocultural. In fact, they vary considerably not only between countries but also between different communities (23, 25). For instance, in Burkina Faso, factors such as age, distance from the household to the primary health center, prenatal visits, previous experience of giving birth at home, negative experiences with health centers, fear of cesarean delivery, and lack of transport, were key predictors of home births (23). To face this situation from an equity perspective, communities with the lowest utilization levels should be prioritized and the access barriers specific to that community identified.

## Discussion

This systematic review set out to examine the influence of UHC on equitable access to healthcare. Study findings indicated that the UHC through the coverage of indigents by the health insurance increased the access to the primary health services but that the quality was not necessarily at the rendezvous. Access to quality care is the foundation of a health system's performance. Equity is respected only if both conditions are met. Indeed, the lack of health professionals and their unequal distribution throughout the country, the stock-outs of medicines and health care equipment, as well as the late payment of rebate payments to health professionals, led to a poor takeover of patients and demotivation of health professionals. Health system governance is the key element and some African countries such as Ghana and Rwanda are success stories in this area as they have a strategic plan for the governance of their health systems according to WHO standards. In addition, other barriers such as ethnicity, religion, physical accessibility (distance from the household to the primary health center), decision-making, gender and autonomy, information and education, age, prenatal visits, previous experience of giving birth at home, negative experiences with health centers, fear of cesarean delivery, and lack of transport impede the utilization of health services and therefore equity.

The first element of equity that is access to health care (the second element being the quality of care) is not respected for different reasons beyond the UHC. Indeed, a systematic review published in 2017 on access barriers to obstetric care at health facilities in sub-Saharan Africa shows that access to obstetric care is riddled by several demand-side barriers including household income, non-availability of means of transportation, indirect transport costs, lack of information on health care services and providers, stigmatization, women's self-esteem, lack of birth preparation, cultural beliefs, and ignorance about required obstetric health services (27). The review also identified supply side barriers, including cost of services, physical distance to health facilities, long waiting times, poor staff knowledge and skill, poor referral practices and poor staff interpersonal relationships (27). Another systematic review analyzing non-financial barriers to access to health services in Ghana, Bangladesh, Vietnam and Rwanda presented perceptions of the condition, home management and local treatment, the influence of family and community, lack of autonomy and agency to act, physical accessibility, and health facility and biomedical barriers are deterrents to access to maternal, neonatal and child health services (28). Non-financial barriers have different expressions and weight depending on context and constitute significant constraints to the equitable access of the full range of health services included under UHC policies.

Studies from developed countries found that despite systems of universal coverage, there was greater inequity, in terms of wait times and receiving of services, for programs deemed non-urgent, elective or for which there will minimal defined treatment protocols (29).

In both developing and developed countries, inequity arose through disparities in quality of care and accessibility of specialized facility-based services. The type of health facilities being accessed varied by different socioeconomic groups. The privileged received most care services in provincial or general hospitals and private clinics, while the underprivileged tended to receive care from the lowest level providers, health centers (29). The underprivileged also had less options regarding providers, receive poor referrals and thereby a restricted set of benefits. This is consistent with findings amongst the underprivileged in the included studies of this review.

The relationship between UHC and equity is evident to the extent that it allows for an increase in health care coverage and health outcomes, but it depends on the availability, accessibility, and capacity of health workers to deliver quality people-centered integrated care. It is therefore important to focus on the primary health care workforce because this is the most cost-effective way to ensure access to primary health care for all (30). To this, the addition of barriers of access to care and good governance of the health system should allow equity in access to quality health care and thus the achievement of the UHC and consequently the SDG 3.

In December 2018, the WHO Health Workforce Director stated: “Health Workers bring us closer each day to achieving UHC. Their rights must be protected in the Health For All movement” (31). In fact, health systems can only function with health workers; improving health service coverage and realizing the right to the enjoyment of the highest attainable standard of health is dependent on their availability, accessibility, acceptability and quality (31, 32). African countries need to have a regional road map that defines actions for scaling up health workforce capacity. Currently, of the 46 countries in the Region, 36 have critical shortage of Human Resources for Health (HRH), 8 with only about 0.8 physicians, nurses and midwives per 1,000 population while the minimum acceptable density threshold is 2.3 per 1,000 population (32). When all categories of health worker are included, the shortfall is estimated at 1.4 million (32). The projected shortage of health worker for the African region will be 18 million by 2030. The reasons of this shortage of health workers are: migration of qualified health workers; inadequate remuneration and incentives; maldistribution of the available health workers significant disparities between rural and urban areas, with shortages in the rural areas (32). Over 90% of pharmacists and dentists practice in urban areas. The situation is the same for medical specialists (86%), general physicians (63%), and nurses and midwives (51%) serve mainly in urban areas; underinvestment in the production of sufficient health workers; inadequate capacity of HRH departments to carry out the main HRH functions; and low implementation of most of the existing plans are identified as the main causes of the present situation that constitutes a key impediment to meeting the needs for health care delivery for all.

It is important for each country to research and identify these uncertainties in each community of the territory. In addition, there is a pressing need for further research on how these specific barriers interrelate and what their role and contribution is to accessing healthcare across different at-risk groups. Then, it will be necessary to put in place adapted strategies to fix these issues, and thus to aspire to increase the efficiency of UHC, which will result in a positive impact on health outcomes. This involves important political decisions. As a first step, African countries should have increased their health budget to reach the 15% provided for in the Abuja Declaration (33). Furthermore, it is important to improve the efficient use of scarce financial resources by enhancing value for money in health. In this regard, it is important for countries to continue to engage in policy dialogue to improve the efficiency and inclusiveness of service delivery, building on on-going operations and gains of the Value for Money Program. Mainstreaming the value for money agenda in all its health operations and contemplating similar action in education is necessary (34). The private sector through public-private partnership could be involved to address quality, efficiency, and financing issues in the health infrastructure and service delivery (34).

## Limitations

There are limited studies on UHC in Africa' only 271 studies on the effects of UHC on equitable access to care were found through the databases. The review has only included 12 studies, which can be seen as low for this topic. However, the term UHC is new and many countries in the continent are still in the early stages. It is an emerging goal in most African countries and therefore still at the implementation stage. A major reason for the low amount of studies was because the authors sought studies that mentioned the term UHC, since using other terms, such as health insurance, would have complicated the analysis as there are several types of health insurance. The other major reason was the focus on underprivileged populations, which was justified in this review as underprivileged populations experience inequity to the largest extent. Additionally, many of these included studies were conducted in Ghana (6). As a result of these limitations, the results of this review cannot be feasibly generalized throughout the continent. The textual narrative synthesis method also has limitations. It is a relatively young method of evidence synthesis, with limited guidance on the conduct of the synthesis. As a result, complete transparency is an inherent limitation of the method. Implementation of tools and techniques to report findings relied on the authors' discretion of best practice, making it difficult for audiences to scrutinize authors' judgements and decisions. Nevertheless, the findings of this review are innovative, and the review is the first to help synthesize evidence on UHC and equitable access in Africa amongst underprivileged populations.

## Conclusion

Universal health coverage and SDG 3.8 cannot be adequately achieved without equitable access to quality care by all citizens, including those who are underprivileged. African countries need well-functioning health systems and governance with sufficient and equitable distribution of health professionals who are adequately trained and skilled to provide quality care to patients. A system to control medicines and prevent material shortages and a strong regulation of the financial system for rebate payments to health professionals is also recommended. Through the increase of coverage by health insurance schemes, there can be improvements in access to care and thereby positive health outcomes in African populations. These requirements will need budget allocation to health from African governments. Lastly, as developing countries attempt to fully implement UHC for all residents, further research is required to assess the underlying changes in equity.

## Author Contributions

NAS and SY conceived of the study and participated in its design and coordination. NAS, AWF, and SY drafted the manuscript. Each author critically reviewed the manuscript for its intellectual content. All authors read and approved the final manuscript.

### Conflict of Interest Statement

The authors declare that the research was conducted in the absence of any commercial or financial relationships that could be construed as a potential conflict of interest.

## Abbreviations

PICO, Population, Intervention: Comparison and Outcomes
UHC: Universal Health Coverage
WHO: World Health Organization
HRH: Human Resources for Health.
