# Supplementary material for: Universal Health Coverage and Facilitation of Equitable Access to Care in Africa
Source: Front Public Health. 2019 Apr 26;7:102. doi: 10.3389/fpubh.2019.00102 (PMC6497736; doi:10.3389/fpubh.2019.00102)
Supplement: Supplementary file 1 [file Data_Sheet_1.docx]

### Appendix 1. Search strategy for each database

Appendix A: Details of the Search strategy

Ovid MEDLINE search strategy: March 6th, 2018

Search for: limit 11 to (yr="2005 -Current" and (english or french))

Results: 50

Database: Ovid MEDLINE(R) Epub Ahead of Print, In-Process & Other Non-Indexed Citations, Ovid MEDLINE(R) Daily and Ovid MEDLINE(R) <1946 to Present>
Search Strategy:
--------------------------------------------------------------------------------
1     universal health coverage.ti,ab,kw. (1213)
2     Universal Coverage/ (2774)
3     exp Health Services Accessibility/ (98579)
4     ((health or medical*) adj3 (care or service*) adj3 access*).ti,ab,kw. (17306)
5     ((health or medical*) adj3 equity).ti,ab,kw. (3025)
6     1 or 2 (3514)
7     3 or 4 or 5 (111117)
8     exp Africa/ (230850)
9     (Algeria or Angola or Benin or Botswana or Burkina Faso or Burundi or Cameroon or Cape Verde or Central African Republic or Chad or Democratic Republic of Congo or Republic of Congo or Cote d'Ivoire or Djibouti or Egypt or Equatorial Guinea or Eritrea or Ethiopia or Gabon or Gambia or Ghana or Guinea or Guinea Bissau or Kenya or Lesotho or Liberia or Libya or Madagascar or Malawi or Mali or Mauritania or Mauritius or Morocco or Mozambique or Namibia or Niger or Nigeria or Reunion or Rwanda or "Sao Tome and Principe" or Senegal or Seychelles or Sierra Leone or Somalia or South Africa or South Sudan or Sudan or Swaziland or Tanzania or Togo or Tunisia or Uganda or Zambia or Zimbabwe).ti,ab,kw. (295236)
10     8 or 9 (394162)
11     6 and 7 and 10 (121)
12     limit 11 to (yr="2005 -Current" and (english or french)) (119)

Ovid EMBASE and CINAHL search strategy: March 6th 2018

| 1. universal health coverage.ti,ab,kw. |
| --- |
| 2. health equity/ |
| 3. ((health or medical*) adj3 (care or service*) adj3 access*).ti,ab,kw. |
| 4. ((health or medical*) adj3 equity).ti,ab,kw. |
| 5. 2 or 3 or 4 |
| 6. exp Africa/ |
| 7. (Algeria or Angola or Benin or Botswana or Burkina Faso or Burundi or Cameroon or Cape Verde or Central African Republic or Chad or Democratic Republic of Congo or Republic of Congo or Cote d'Ivoire or Djibouti or Egypt or Equatorial Guinea or Eritrea or Ethiopia or Gabon or Gambia or Ghana or Guinea or Guinea Bissau or Kenya or Lesotho or Liberia or Libya or Madagascar or Malawi or Mali or Mauritania or Mauritius or Morocco or Mozambique or Namibia or Niger or Nigeria or Reunion or Rwanda or "Sao Tome and Principe" or Senegal or Seychelles or Sierra Leone or Somalia or South Africa or South Sudan or Sudan or Swaziland or Tanzania or Togo or Tunisia or Uganda or Zambia or Zimbabwe).ti,ab,kw. |
| 8. 6 or 7 |
| 9. 1 and 5 and 8 |

Global health search strategy: March 9th 2018

"universal health coverage" AND access* AND africa*

Publication year limit: 2005-2018

Document type: Journal article, and conference papers

Results: 98 references found.

**Appendix 2.** Characteristics of excluded studies after full review (did not meet the inclusion criteria)

| **First Author, Year** | **Title** | **Reason for Exclusion** |
| --- | --- | --- |
|  |  |  |
| **Harris et al. (2011).** | "Inequities in access to health care in South Africa." | Coverage is not governmental |
| **Lagomarsino, G., Garabrant, A., Adyas, A., Muga, R., Otoo, N. (2012)** | "Moving towards universal health coverage." | Non specific to Africa, and about the policy process and implementation |
| **Mtei, G., Makawia, S., Masanja, H. (2014).** | "Monitoring and Evaluating Progress toward Universal Health Coverage in Tanzania" | Narrative review |
| **Sieleunou, I. (2011).** | "Health worker migration and universal health care in Sub-Saharan Africa" | Narrative review |
